# Antenna Design and Construction of a Free-Pass System for Hi-Pass Type Ticket Gates in Subways for Transportation-Disadvantaged Individuals

**DOI:** 10.3390/s25010005

**Published:** 2024-12-24

**Authors:** Yunsub Lee, Cholu Lee, Cheol Yoon, Woosu Kim

**Affiliations:** Graduate School of Convergence Technology and Energy, Tech University of Korea, Siheung-si 15073, Republic of Korea; skua1204@tukorea.ac.kr (Y.L.); martinlee2k@tukorea.ac.kr (C.L.)

**Keywords:** microstrip antenna, patch antenna, Hi-pass, free-pass system, transportation vulnerable

## Abstract

This paper examines the design of antennas for Hi-pass type turnstiles needed to implement a subway free-pass system targeting transportation-disadvantaged individuals. The subway free-pass system allows individuals who have a free-pass card to approach the turnstile with the card on their person, which opens the gate automatically. This system, like the highway Hi-pass, allows users to pass through the subway gate without needing to scan a ticket. For the system to function, antennas are required at both the entrance and exit gates, with an additional antenna needed for the free-pass card, totaling three antennas. The free-pass card functions as a radio-frequency identification (RFID) tag, while the system uses Bluetooth communication. In this paper, we propose a square patch-type microstrip antenna that can be integrated within the turnstile, performing excellently in the Bluetooth band range of 2.420 to 2.485 GHz.

## 1. Introduction

Railways and subways have rapidly adopted the latest communication technologies to enhance safety, improve operational efficiency, and provide better services to passengers. Wireless communication-based train-to-ground systems are being actively researched for advanced railway services, including automatic train control, real-time onboard video surveillance, real-time onboard monitoring, remote train diagnostics, and onboard Internet access for passengers [1,2,3,4]. However, public transport is often difficult for individuals with physical impairments or mental health challenges to use. Traditional urban public transport systems generally do not provide effective access services for people with disabilities, particularly for disabled, wheelchair, and blind (DWB) passengers [5,6]. Traditional urban public transportation systems worldwide are generally designed for the healthy population and rarely consider the needs of people with disabilities. Currently, most fare payment systems for public transportation combine near-field communication (NFC) and mobile payment technologies. However, studies are examining gate-free technology that allows access without tag action, using face recognition and object tracking [7,8].

Railway stations, standardized with waiting rooms and platforms, have structural and facility limitations that inevitably lead to congestion due to the current physical fare payment system that requires tagging. By introducing non-contact fare payment technology, it is possible to enhance user convenience when moving and transferring, reduce congestion in bottleneck areas around toll gates, and shorten transfer distances via architectural improvements [9]. A gate-free pass system is a non-contact fare payment system that eliminates the need for toll gates. Such systems also increase safety. A gate-free pass system can help alleviate congestion in bottleneck areas around toll gates and minimize contact with facilities, thereby reducing inconvenience for people with disabilities [10]. The subway free-pass system allows transportation-disadvantaged individuals with a gate-free pass card in their possession to approach the gate, which then opens automatically. This enables passage through the subway gate without the need to stamp a ticket, similar to the highway Hi-pass system [11]. To implement a gate-free pass system, antennas are needed at both the entrance and exit gates, with an additional antenna for the free-pass card, for a total of three antennas. The free-pass card functions as an RFID tag [12,13], and the system operates using Bluetooth Low Energy (BLE) communication [14,15].

This paper proposes a directional antenna for locating mobile terminals within the gate-free pass system. We introduce a stacked air-gap patch antenna that meets BLE performance requirements and offers high directivity, tailored for the gate-free pass system. The proposed antenna was optimized through simulation, and its excellent performance was verified after fabrication and measurements. An air gap was incorporated between the patch radiator and ground plane to enhance bandwidth. Furthermore, the antenna performed outstandingly and had a high recognition rate during testing at the Hi-pass gate of Daejeon Jungang-ro Station in South Korea. In addition, the pilot operation was conducted at Panam Station (Daejeon University) and Banseok Station in Daejeon, South Korea, from November to December 2022. Since January 2023, the system has been fully operational across all Daejeon subway stations.

## 2. Gate-Free Pass System

**Figure 1:**
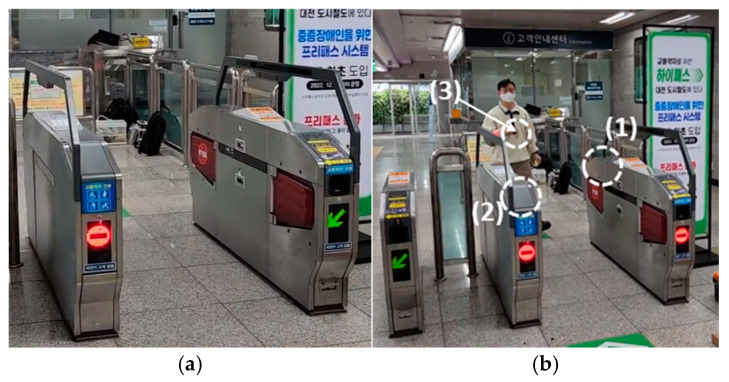
Configuration of the gate-free pass system: (**a**) typical gate entrance and (**b**) gate-free pass entrance; (1) entrance gate (2) exit gate (3) free-pass card.

The gate-free pass system uses mobile, beacon, and BLE technologies to allow vulnerable passengers to pass through automatically when they possess a free-pass card. Figure 1a shows a typical gate entrance in the gate-free pass system.

**Figure 2:**
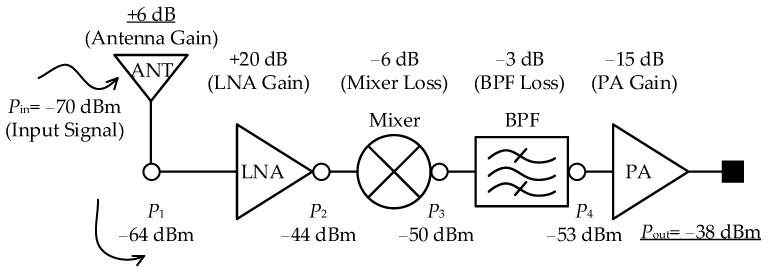
Block diagram of BLE RF chain.

The gate-free pass system requires antennas at both the entrance and exit gates, as well as one for the free-pass card, totaling three antennas. In this study, antennas for the entrance and exit gates were designed and manufactured, and commercial free-pass cards were used to evaluate the gate-free pass system. As shown in Figure 1b, the gate-free pass system consists of the entrance gate (1), exit gate (2), and free-pass card (3) antennas. The system operates through communication between the terminal and server once the free-pass card detects the signal from the BLE antenna at the gate. Figure 2 shows the BLE RF chain of the proposed free pass system, where the gain and power of each component are calculated. This BLE RF chain receives the input signal and then amplifies, converts, and filters it through each component to ultimately generate the output signal. The input signal strength is calculated based on the worst-case scenario of −70 dBm, considering the path loss. The main calculation involves adding or subtracting the gain or loss at each stage to determine the signal strength. As a result, the final output must reach −38 dBm or higher for the system to operate effectively, which requires the antenna gain to be at least 6 dB. The gain of the proposed antenna, which is 8 dB or higher, is sufficient for the system to function as intended.

## 3. Antenna Design

**Figure 3:**
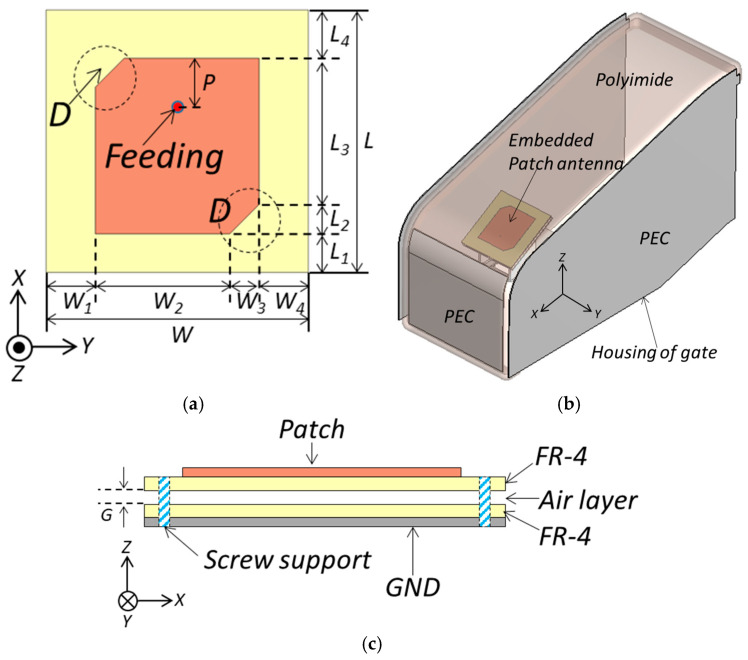
Configuration of the proposed antenna: (**a**) top view, (**b**) antenna installed inside the gate housing, and (**c**) side view.

Figure 3 shows the configuration of the proposed antenna, which consists of a modified rectangular patch, two FR-4 (epoxy) substrates, and a ground plane. The modified rectangular patch is printed on a 1 mm FR-4 substrate with a relative dielectric constant of 4.4 and a loss tangent of 0.02. The rectangular patch is positioned at the center of the FR-4 substrate, with the feed position optimized to adjust the input impedance of the antenna and improve bandwidth. A broadband characteristic is achieved by incorporating an air layer (*G*) between the two FR-4 substrates.

The proposed antenna was simulated and optimized using the commercial software CST Studio Suite 2024. The optimized parameters were as follows (units, mm): *W* = 63.5, *W*_1_ = 11.9, *W*_2_ = 32.7, *W*_3_ = 7, *W*_4_ = 11.9, *L* = 63.5, *L*_1_ = 11.9, *L*_2_ = 7, *L*_3_ = 32.7, *L*_4_ = 11.9, *P* = 12, and *G* = 2.4. The patch measures 39.7 × 39.7 mm^2^, with a height of less than 5 mm. For efficient communication, the patch antenna is positioned at a 30-degree angle relative to elevation. To achieve circular polarization (CP), the corners of the patch are trimmed, as shown in part *D* of Figure 3a. CP is advantageous in Bluetooth applications as it helps suppress multipath interference and reduces polarization mismatch. To meet system requirements requires an antenna with high directivity covering the BLE band of 2.420 to 2.485 GHz within a gate housing, as shown in Figure 3b. It is also necessary to analyze the antenna performance within the gate structure. Considering these requirements, a stacked air-gap patch antenna is proposed, as illustrated in Figure 3c. With a stacked patch structure, shown in Figure 3a, the optimal peak gain can be achieved using the FR-4 substrate. In this paper, we propose an antenna using an FR-4 substrate with a single permittivity that meets the bandwidth and gain requirements for the gate-free pass system within physical constraints. The effects of the air gap on bandwidth and the patch angle on directivity are analyzed to optimize performance.

## 4. Simulated and Measured Results

### 4.1. Parametric Study

**Figure 4:**
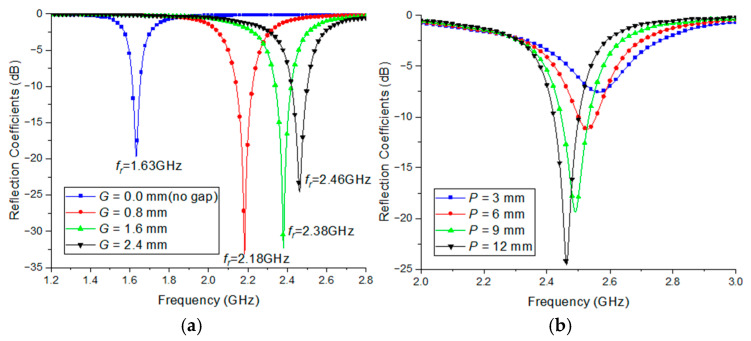
Comparison of reflection coefficients: (**a**) variation in air-gap of substrate (*G*) and (**b**) different feed positions (*P*).

Using simulations, parametric studies analyzed the effects of the air gap (*G*) and feed position (*P*) on the reflection coefficients. Figure 4a shows the resonant frequency as a function of changes in the air gap between the patch radiator and ground (*G*). This shows that as *G* increases from 0.8 to 2.4 mm, the resonant frequency shifts from 1.63 to 2.46 GHz. This occurs because an increase in *G* results in a decrease in inductance. Moreover, an air gap of 2.4 mm achieves the maximum bandwidth within the BLE bands. The width and length of the radiator, substrate thickness, air gaps, and feed positions were varied incrementally, with the bandwidth calculated at each step until an optimal maximum bandwidth for broadband use was achieved. Figure 4b presents the simulated reflection coefficients of the proposed antenna with *p* values of 3, 6, 9, and 12 mm. This demonstrates that the impedance of the antenna can be adjusted by varying the feed position, *P*. In this case, the optimal values for *G* and *P* were determined to be 2.4 mm and 12 mm, respectively.

**Figure 5:**
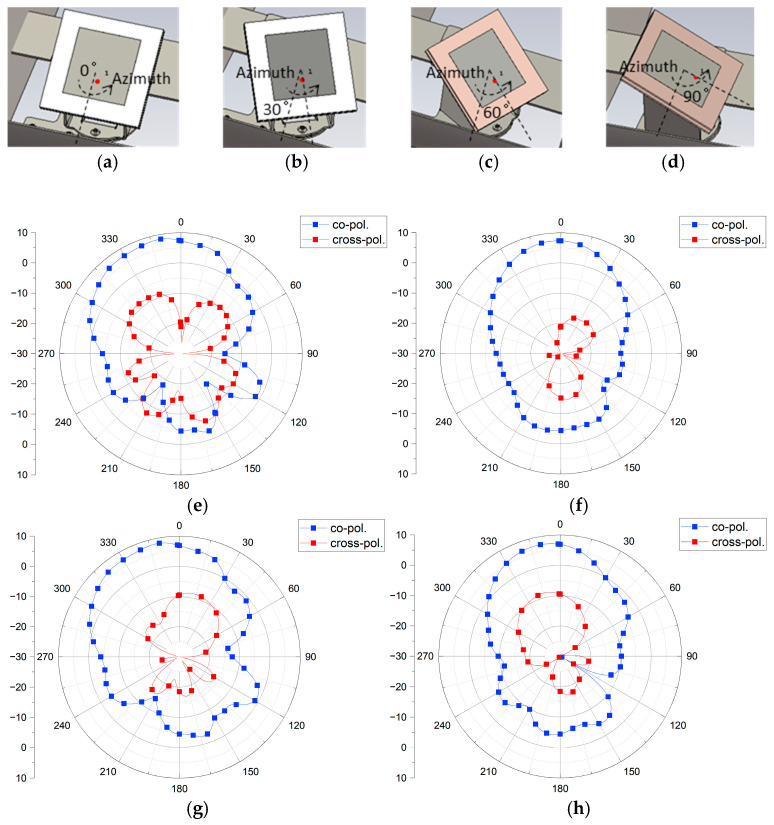

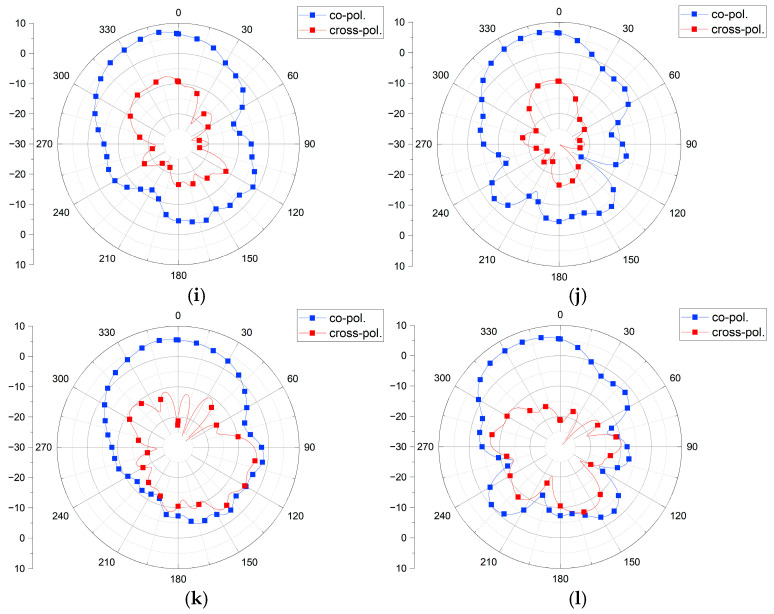
Simulated radiation pattern characteristics at 2.42 GHz with varying azimuth angles: (**a**) radiation pattern at 0-degree azimuth angle, (**b**) radiation pattern at 30-degree azimuth angle, (**c**) radiation pattern at 60-degree azimuth angle, (**d**) radiation pattern at 90-degree azimuth angle. Corresponding planes for each azimuth angle: (**e**) XZ-plane for (**a**) 0-degree azimuth, (**f**) YZ-plane for (**a**) 0-degree azimuth, (**g**) XZ-plane for (**b**) 30-degree azimuth, (**h**) YZ-plane for (**b**) 30-degree azimuth, (**i**) XZ-plane for (**c**) 60-degree azimuth, (**j**) YZ-plane for (**c**) 60-degree azimuth, (**k**) XZ-plane for (**d**) 90-degree azimuth, and (**l**) YZ-plane for (**d**) 90-degree azimuth.

Figure 5 shows the radiation patterns for various patch angles in the azimuth direction while keeping the elevation angle fixed at 30-degrees. The simulations were conducted by setting the angles in Figure 5a–d to 0, 30, 60, and 90-degrees, respectively.

Figure 5e,f show the radiation patterns in the XZ-plane and YZ-plane, respectively, when the patch angle is 0-degrees in the azimuth direction (as in Figure 5a). Similarly, Figure 5g,h show the radiation patterns when the patch angle is set as in Figure 5b, Figure 5i,j show the patterns for the patch angle in Figure 5c, and Figure 5k,l present the patterns for the patch angle in Figure 5d. When the patch angle is set to 30-degrees (Figure 5b), the radiation pattern in the XZ-plane (Figure 5g) has a peak gain of 8.27 dBi with a main lobe direction of 11-degrees, matching the characteristics in Figure 5e. The YZ-plane radiation pattern (Figure 5h) shows a peak gain of 7.4 dBi and a main lobe direction of 9-degrees, which are the best characteristics among all angles tested. The main lobe direction of the radiation pattern should be set to ensure recognition at a distance of at least 1.5 m when a transportation-vulnerable individual approaches the gate. Therefore, the optimal patch angle for efficient communication when such individuals pass through the gate is an azimuth angle of 30-degrees. Based on these results, an experiment was conducted at a 30-degree setting, and a field test was performed.

**Figure 6:**
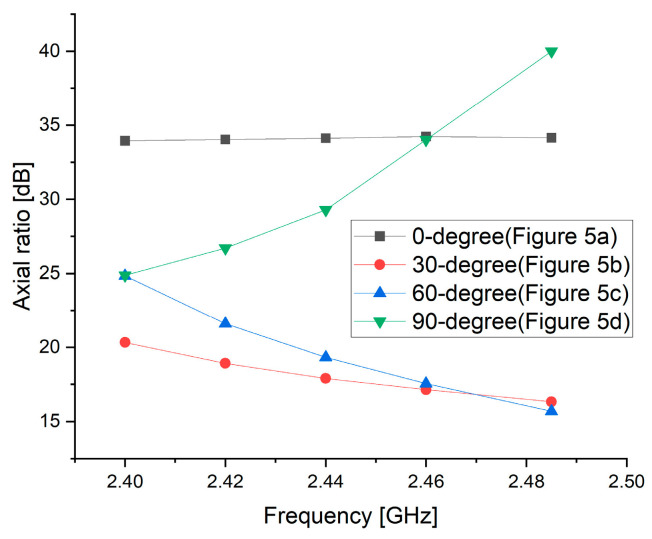
Axial ratio characteristics of the proposed antenna.

Figure 6 illustrates the simulated axial ratio at various incidence angles (0°, 30°, 60°, and 90°) within the frequency range of 2.40 GHz to 2.48 GHz. As shown in Figure 6, the axial ratio decreases notably at 30° and 60°, indicating improved polarization characteristics at these angles. Specifically, the minimum axial ratio of approximately 15 dB is observed near 2.48 GHz, suggesting polarization approaching circular. For systems requiring circular polarization, the axial ratio must generally be reduced to below 3 dB. In this study, the linear polarization characteristics of a conventional patch antenna are converted to elliptical polarization to enhance system efficiency.

### 4.2. Experimental Results

**Figure 7:**
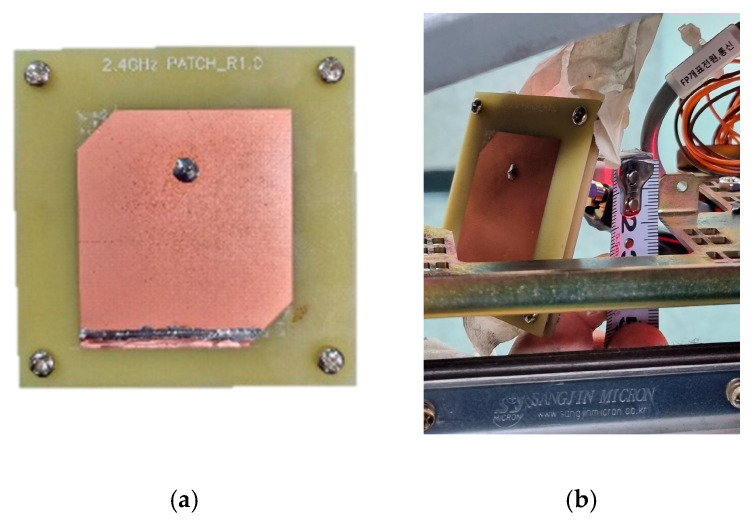

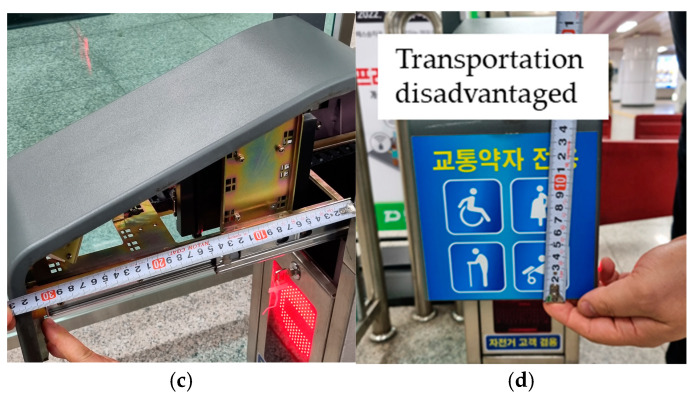
Prototype and setup of the patch antenna: (**a**) prototype of the proposed antenna, (**b**) patch antenna located in the gate housing, (**c**) side view of the gate housing, and (**d**) front view of the gate housing.

Based on the optimized dimensions from the simulation, the proposed rectangular patch antenna was fabricated, as shown in Figure 7a. Figure 7b shows the patch antenna positioned inside the actual gate housing, mounted approximately 3 cm above the horizontal plane. Figure 7c is a side view of the gate housing, which is 33 cm long, while Figure 7d shows a front view with a height of 17 cm. The patch antenna is angled at 30 degrees to the horizontal, facing the entrance. The detailed experimental setup within the subway system is as follows. In the experiment, a user carrying a free pass card embedded with a BLE tag passes through the gate. The system’s recognition rate is evaluated based on the BLE tag’s distance, direction, and speed relative to the gate. Furthermore, the proposed antenna is mounted on the gate, and its signal quality is assessed across various distances and angles. The feasibility of the proposed antenna was validated through field tests conducted in a real-world environment, with particular attention to vulnerable road users.

**Figure 8:**
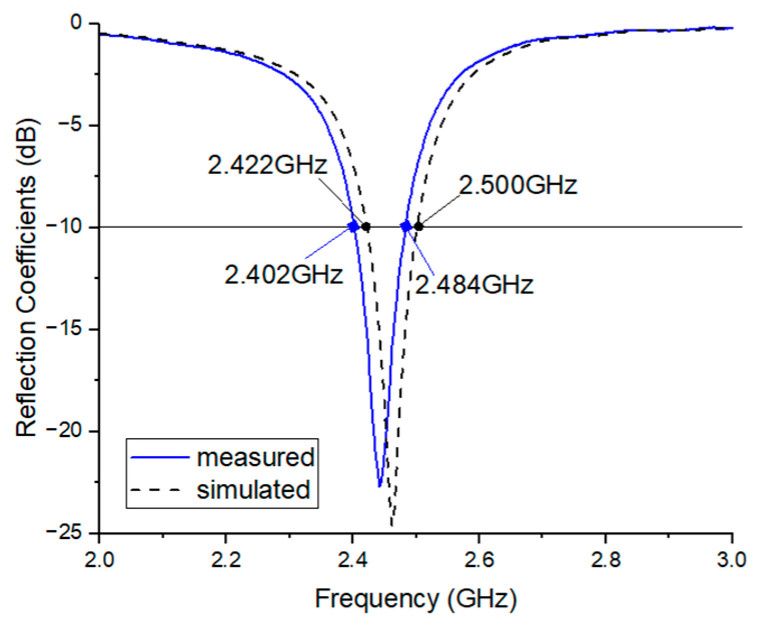
Simulated and measured reflection coefficients of the proposed antenna.

Figure 8 shows the simulated and measured reflection coefficients of the proposed antenna. As illustrated, the simulation results indicate a bandwidth of 78 MHz, ranging from 2.422 to 2.500 GHz, with a reflection coefficient of −24.6 dB at the resonance point of 2.462 GHz, based on a reflection coefficient of −10 dB (VSWR 2:1). The measured results give a bandwidth of 82 MHz, from 2.402 to 2.484 GHz, with a resonance point at 2.442 GHz and a reflection coefficient of −22.6 dB. Thus, the antenna satisfies all BLE bands (2.420 to 2.485 GHz) and has excellent measured results consistent with the simulation predictions.

**Figure 9:**
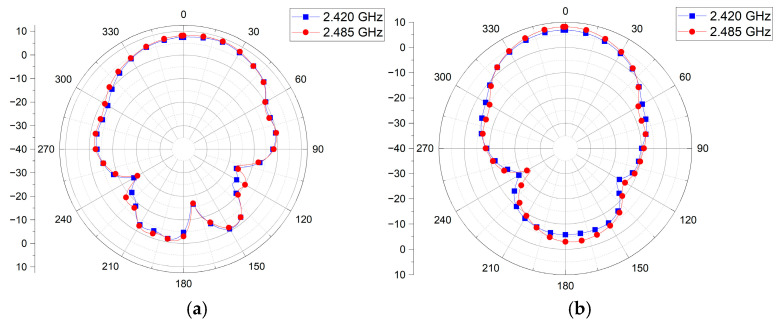
Measured radiation patterns of the proposed antenna: (**a**) XZ-plane and (**b**) YZ-plane.

Figure 9 presents the measured radiation patterns of the antenna in the XZ-plane (E-plane) and YZ-plane (H-plane) at frequencies of 2.420 and 2.485 GHz, within the operating frequency band. Figure 7a,b show that the peak gain is 8.34 dBi at 2.420 GHz and 8.69 dBi at 2.485 GHz.

**Figure 10:**
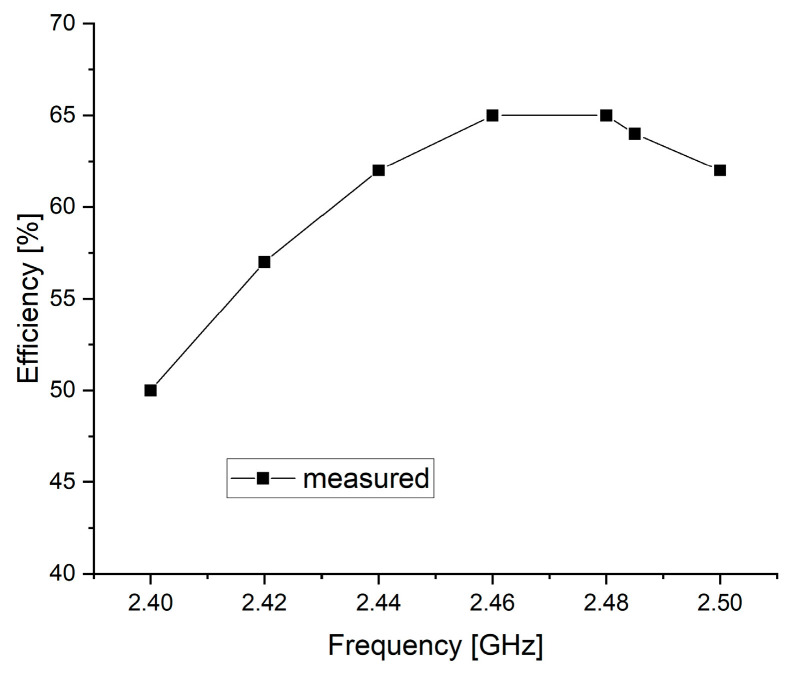
Measured radiation efficiency of the proposed antenna.

Figure 10 shows the measured radiation efficiency of the proposed antenna across the 2.400–2.500 GHz range. The measured radiation efficiency of the patch antenna is 50% at 2.400 GHz, 57% at 2.420 GHz, 62% at 2.440 GHz, 65% at 2.460 GHz, 65% at 2.480 GHz, 64% at 2.485 GHz, and 62% at 2.500 GHz. The maximum efficiency is at 2.460 and 2.480 GHz, which is sufficient for BLE applications.

Table 1 compares the measured characteristics of the proposed antenna from 2.400 to 2.500 GHz and shows the peak gain and main lobe direction for each frequency. The table shows that at 2.460 GHz, the peak gain is 8.814 dBi, and the main lobe direction is 19-degrees.

## 5. Conclusions

In this paper, we propose a high-directivity rectangular patch antenna for a gate-free pass system designed to assist transportation-vulnerable individuals. The proposed antenna is engineered to have high directivity, enabling transportation-vulnerable users to recognize the position of the free-pass card when passing through the gate. The performance of the proposed antenna at different angles was verified, showing that it performs optimally at a 30-degree angle for distinguishing adjacent free-pass cards. Given the excellent performance of the antenna, subway gate-free pass systems could be implemented in various countries, helping to reduce the inconvenience faced by transportation-vulnerable individuals.

## Figures and Tables

**Table 1:** Comparison of the measured characteristics of the proposed antenna.

Frequency (GHz)	Peak Gain (dBi)	Main Lobe Direction (degree)
2.400	7.853	18.0
2.420	8.347	19.0
2.440	8.677	19.0
2.460	8.814	19.0
2.480	8.742	19.0
2.500	8.452	19.0

## Data Availability

The data that support the findings of this study are available from the corresponding author upon reasonable request.
